# Development of a highly efficient ion-ozone cavitation technology for accelerated bread production

**DOI:** 10.1038/s41598-021-98341-w

**Published:** 2021-09-27

**Authors:** Sholpan Tursunbayeva, Auyelbek Iztayev, Aizhan Mynbayeva, Mariam Alimardanova, Baurzhan Iztayev, Madina Yakiyayeva

**Affiliations:** 1 Almaty Technological University, 100 Tole be Street, Almaty, 050012 Kazakhstan; 2 M.Kh. Dulaty Taraz State University, 60, Tole bi str., Taraz, 080000 Kazakhstan

**Keywords:** Biotechnology, Engineering

## Abstract

The bakery market is one of the most capacious in Kazakhstan. Manufacturers of bread products are in dire need of the introduction of intensive technologies for improving product quality and safety. This article presents the results of research to develop technology for accelerated production of bread with ion-ozone cavitation treatment. The influence of various modes of exposure to ion-ozone cavitation has been investigated. After baking, bread samples were examined for organoleptic, physicochemical, rheological and microbiological indicators. The optimal method is treatment with ion-ozone at a concentration of 0.0025 units/mg, at a pressure of 1.0 atm for 1 min. As a result, it was proved that this mode accelerates the process of obtaining dough and shortens the fermentation time, and baking bread increases the qualitative and quantitative indicators according to the control method. The results showed that the ion-ozone technology reduces the length of the process of making dough and bread by three times compared to traditional technologies. The developed products with existing analogues in the Kazakhstan market will differ due to their high taste and consumer properties, product safety, long shelf life and low cost.

## Introduction

Solving the problem of healthy human nutrition is one of the most important tasks of our time. Grain processing products meet the requirements of good nutrition as well as possible. In this regard, there is a need to create a wide range of new grain products that allow the rational use of all valuable natural components, with a significant reduction in production costs^1,2^.

That is why, in the practice of grain processing, considerable attention is paid to the introduction of progressive methods and high-performance equipment in order to increase the efficiency of using grain during its processing^3,4^.

Currently, the issue of expanding the range of bakery products remains relevant. The primary role is played by increasing the taste and nutritional properties of bread while maintaining its low price. This is achieved by improving the technology of baking by changing the parameters of grain preparation, the degree and method of grain grinding, the variety of recipes due to the inclusion of other grains and other components during kneading, and improving the technology for loosening the dough and the conditions for baking bread^5,6^.

All over the world there is a generally accepted international classification of wheat quality, which systematizes grain crops in various aspects. According to this systematization, grain, depending on various indicators, is divided into six main classes. The first three classes (I, II and III) belong to valuable varieties of wheat and are used in the flour-grinding and baking industry^7–9^. Also, the grain of this group is exported. We used grade 3 wheat in our research work.

Scientists have investigated kneading dough in a vacuum^10^ and in an atmosphere of air^11,12^, oxygen^13^, nitrogen^14,15^, hydrogen^16^ and carbon dioxide^17^. Tests have shown that significant amounts of gas from the atmosphere in which kneading takes place can be mechanically trapped (occluded) in the dough. It has been established that if the dough is kneaded in an atmosphere of air enriched with oxygen, then the gas bubbles formed in the dough during kneading are a factor of the oxidative effect of oxygen on the corresponding components of the dough, especially on its protein–proteinase complex^18,19^.

However, none of these methods satisfy the producers of bread products, complicating the process of making dough and baking bread. Therefore, we propose an accelerated method for preparing bread products using ion-ozone cavitation technology.

The essence of accelerated dough preparation methods is the intensification of microbiological, colloidal and biochemical processes occurring during dough maturation, as a result of:

- enhanced mechanical processing of the dough during kneading;
- the use of acidifying or activated semi-finished products;
- increasing the temperature of the dough;
- increasing the dosage of biological disintegrants.

The advantages of accelerated methods are the reduction to a minimum of the number of containers for dough fermentation, the possibility of operating enterprises in two shifts and with a part-time working week, a reduction of flour costs during fermentation, improvement of production culture, etc.^20,21^.

In recent years, ozone, ions, ozone and ion-ozone technologies, which have a number of advantages over special additives and technologies, have been increasingly used in the food industry. Ozone treatment is used to treat grain crops and their processed products for the purpose of disinfecting and extending the shelf life^22–25^. Ionic treatment is used to disinfect and purify water^26,27^. The use of ion-ozone technology agents with many useful properties (bactericidal, redox, etc.) in food production is the latest trend and is a promising direction in food production. For example, it was used to disinfect legumes, oilseeds^28,29^ and sugar beets^30^. Currently, scientists at the Almaty Technological University are conducting research on the use of ozonized, ionized and ion-ozonized water in the production of flour, bakery, pasta and flour confectionery products from wheat flour and flour from a mixture of wheat, grain, oilseeds and legumes that improve the quality, safety and environmental friendliness of the finished products.

One of the promising technologies that provides a significant intensification of production processes, and opens up a wide range of opportunities for expanding the range of grain, bakery and other types of products, is cavitation processing of raw materials, which makes it possible to obtain grain suspensions—products with a certain set of physicochemical and organoleptic properties.

The proposed technology is based on the physical phenomenon of cavitation, which is generated either by ultrasound (acoustic) or hydraulic impulses (rotational). Acoustic cavitation units are already being used in various branches of the food industry.

Literally translated from Latin, cavitation is emptiness, the formation and collapse of microscopic bubbles in a liquid under the influence of external forces. The most famous effect of this phenomenon leads to the destruction of the metal parts of turbines, propellers and other mechanisms operating in water at variable pressure. But it is precisely this that has become a powerful factor in technological transformation in food production^31,32^.

The external manifestation of the phenomenon is that the water subjected to cavitation acquires some of the properties of boiling water, while remaining completely cold. Like boiling water, it becomes a powerful solvent and is able to actively combine with proteins and other natural high-molecular-weight components of agricultural raw materials. But, while remaining cold, unlike real boiling water, such water no longer destroys or changes their natural properties, which is very important for the food industry and medicine^33,34^.

‘Cold boiling water’ has now turned out to be very suitable for the production of feed for farm animals and poultry. Cavitation treatment of water used to moisten the feed mixture improves its digestibility, disinfects it and, surprisingly, increases its total volume. And the cavitation treatment of drinking water for animals during fattening provides an increase in their weight gain, reduces the length of the fattening process itself and sharply reduces disease incidence and mortality of livestock.

In milling production, cavitation treatment of water used to moisten grain sharply reduces the time it takes to prepare it for grinding. The Vologda Bakery team has achieved amazing results in their work using the new technology. With the help of emulsions of cavitation-treated water in vegetable oil, they managed to stop using expensive so-called emulsifiers and baking improvers, which are unsafe for human health, in baking bread. The use of ‘cold boiling water’ in the food industry, among other things, reduces bacterial contamination^35–37^.

The preparation of bakery dough with cavitation-activated water, accompanied by hydration structuring of gluten proteins, makes it possible to increase the specific volume of bread, increase its elasticity, slow down hardening and reduce the use of bakery improvers. The processing of sugar–salt solutions in a cavitation reactor before mixing with the dough makes it possible to reduce the salt and sugar content in bread by 15–20% without changing the taste and nutritional value of the product. Cavitation technology makes it possible to produce fat emulsions for dough only from vegetable fats and water, since in the process of their preparation, partial hydrolysis of fats occurs, with the formation of di- and monoglycerides which are natural emulsifiers^38–40^.

Ion-ozone processing of products affects the biological and physiological effects on development and vital activity; it also produces a disinfecting effect, increases the biological value of bread and increases the shelf life of finished bread by reducing the negative influence of external factors (increasing the safety of grain, reducing factors leading to diseases of bread, etc.) on the storage of ready-made bread^41,42^.

The purpose of this study was to develop a technology for making bread from class 3 wheat using ion-ozone cavitation technology.

## Research results

Currently, this ion-ozone cavitation technology is little used for accelerated dough preparation in the near and far abroad is absent. This dough preparation technology is simple, inexpensive and environmentally friendly. Thus, it was used in this work to accelerate the preparation of dough and bread from it.

The physical and mechanical indicators of the quality of wheat of the 3rd class were determined. The results are presented in Table 1.

**Table 1:** Indicators of the quality of class 3 wheat grain.

Indicator	Result	International research standard
Mass per hectolitre	76 kg/hl	ISO 7971-2
Protein (maximum)	12.5% on a dry basis	ISO 20483
Amount of gluten	23–25%	ISO 21415
Humidity (maximum)	14%	ISO 712
Falling number (Hagberg–Perten method)	200–250 s	ISO 3093

From the data in Table 1 it can be seen that all investigated indicators were in accordance with the requirements of international standards, indicating that the investigated grain meets the requirements. Grade 3 wheat grain was passed through a laboratory mill and sieves with different hole sizes and thus whole flour was obtained, which was used in further research.

Eight flour samples were selected and prepared from class 3 wheat flour by ion-ozone cavitation treatment, which were subjected to three types of exposure: X_0_—control sample, X_1×10_4—ion-ozone cavitation concentration, X_2_—ion-ozone air cavitation pressure, X_3_—processing time, t (min).

The physicochemical parameters of flour obtained from whole-ground class 3 wheat with ion-ozone cavitation treatment were investigated. The results are shown in Table 2.

**Table 2:** Physical and chemical parameters of flour obtained from whole-ground class 3 wheat with ion-ozone cavitation treatment.

No.	Factors				Physical and chemical indicators									
	X_0_	X_1×10_4, unit/mg	X_2_, atm	X_3_, min	y_1_, %	y_2_, %	y_3_, %	y_4_, %	y_5_, %	y_6_, %	y_7_, CFU/g	y_8_, CFU/g	y_9_, %	y_10_, con. units
**Control sample**					6.15	1.51	59.57	1.92	10.75	43.2	12	13	25.0	86.0
1	+	25	2.0	10	6.0	1.52	60.99	1.66	10.46	40.6	21	15	27.0	85.0
2	+	5	2.0	10	6.13	1.52	59.27	1.71	10.39	40.9	38	20	25.4	84.5
3	+	25	1.0	10	6.02	1.5	57.34	1.61	10.11	42.1	23	31	26.0	81.3
4	+	5	1.0	10	6.11	1.5	59.18	1.63	10.22	41.3	23	22	27.2	86.0
5	+	25	2.0	5	6.34	1.53	56.38	1.62	10.22	40.9	36	7	26.7	84.1
6	+	5	2.0	5	6.31	1.58	58.02	1.58	9.56	45.0	2	3	28.0	88.0
7	+	25	1.0	5	6.11	1.50	62.67	1.51	9.35	42.2	24	15	27.4	83.4
8	+	5	1.0	5	6.43	1.54	55.02	1.61	9.1	41.5	19	23	26.8	87.5

y_1_—protein, y_2_—mass fraction of fat, y_3_—carbohydrates, y_4_—ash content, y_5_—mass fraction of fibre, y_6_—whiteness of flour, y_7_—amount of yeast, y_8_—amount of mould, y_9_—amount of gluten, y_10_—deformation of gluten (on the IDK device).

According to Table 2, changes in the indicators of physical and chemical properties can be noted depending on a change in the ion-ozone cavitation treatment mode. So, in comparison with the control sample, which has excellent results in terms of carbohydrate content, ash content, fibre and flour whiteness, options 5, 6 and 8 have the best indicators in terms of the mass fraction of fat and protein and options 1, 2 and 7 are distinguished by the best indicators in terms of carbohydrate content; number 1 also has a good fibre content. Numbers 6 and 7 also differ in that they differ from the rest of the options in the whiteness of flour. Numbers 2 and 5 have a lot of yeast, and number 3 is mouldy, which makes these options unattractive. Numbers 6, 7 and 8 are good in terms of the quantity and quality of gluten. Number 4 does not differ in any parameter. The most attractive among the rest of the options are numbers 6, 7 and 8.

Table 3 shows the rheological properties of the dough made from whole-ground class 3 wheat flour with ion-ozone cavitation treatment. Physicochemical and rheological indicators of quality were investigated. In determining the rheological properties, alveograph, farinograph and Mixolab devices were used (Table 3).

**Table 3:** Rheological properties of dough made from whole-ground class 3 wheat flour with ion-ozone cavitation treatment.

No.	Factors				Alveograph					Farinograph					Mixolab			
	X_0_	X_1×10_4, unit/mg	X_2_, atm	X_3_, min	y_1_, min	y_2_, min	y_3_, N/m^2^	y_4_, N	y_5_, N/m	y_6_, %	y_7_, min	y_8_, min	y_9_, FE	y_10_, min	y_11_, mg/kg	y_12_ unit/l	y_13_, Pa·s	y_14_
**Control sample**					9.4	61	7.44	123	134	66.1	13.8	13.6	10	8.55	16.92	23.28	29.82	44.98
1	+	25	2.0	10	24.4	86	1.11	81	134	66.4	18.0	13.2	14	8.38	16.78	22.75	30.83	45.00
2	+	5	2.0	10	18.2	67	1.63	83	109	66.2	16.5	12.6	15	8.43	16.83	22.98	31.08	45.00
3	+	25	1.0	10	19.2	75	1.3	127	100	66.7	20.0	12.6	9	8.57	16.83	23.18	30.70	45.02
4	+	5	1.0	10	19.6	78	1.35	147	105	66.3	16.7	13.6	12	8.45	16.95	23.45	30.42	45.02
5	+	25	2.0	5	18.4	69	1.22	94	84	66.3	11.2	13.9	17	8.23	16.73	23.10	30.82	45.02
6	+	5	2.0	5	18.2	67	1.3	93	87	65.7	11.0	13.6	18	8.43	16.58	22.77	31.03	45.02
7	+	25	1.0	5	12.9	34	4.5	190	153	66.5	16.9	14.0	13	7.72	16.92	23.28	30.43	45.00
8	+	5	1.0	5	20.8	88	1.14	86	100	66.6	10.7	9.0	4	6.80	16.68	22.78	31.22	45.00

y_1_—dough formation time, y_2_—dough tensile index, y_3_—dough elasticity ratio, y_4_—dough strength, y_5_—dough elasticity indicator, y_6_—water absorption capacity, y_7_—dough formation time, y_8_—dough stability, y_9_—degree of dough softening, y_10_—dough kneading time, y_11_—gluten, y_12_—amylase, y_13_—viscosity, y_14_—retrogradation.

In the process of making white bread from wheat flour, preliminary kneading of the dough takes 9 min, then the dough is kneaded for 18–22 min; the next stage is the first proofing of the dough piece, which is 60–70 min in duration. The dough is kneaded for 20 min, then the second stage of proofing of the dough takes place (70 min), providing an airy and uniform bread structure. Bread baking takes 63–68 min; after baking, the bread is rested for 10–19 min.

From the data in Table 3, it can be seen that the seventh sample has better results compared to the others. Based on the results of the dough quality studies, one can immediately conclude that the optimal option in many respects is number 7. It compares favourably with the others in terms of dough formation time, elasticity-to-extensibility ratio, dough strength, dough elasticity indicator, dough stability, gluten content and dough viscosity. In terms of water absorption capacity and dough retrogradation, all dough variants have similar results. The options less favourably differing from the rest are numbers 2, 5, 6 and 8.

**Figure 1:**
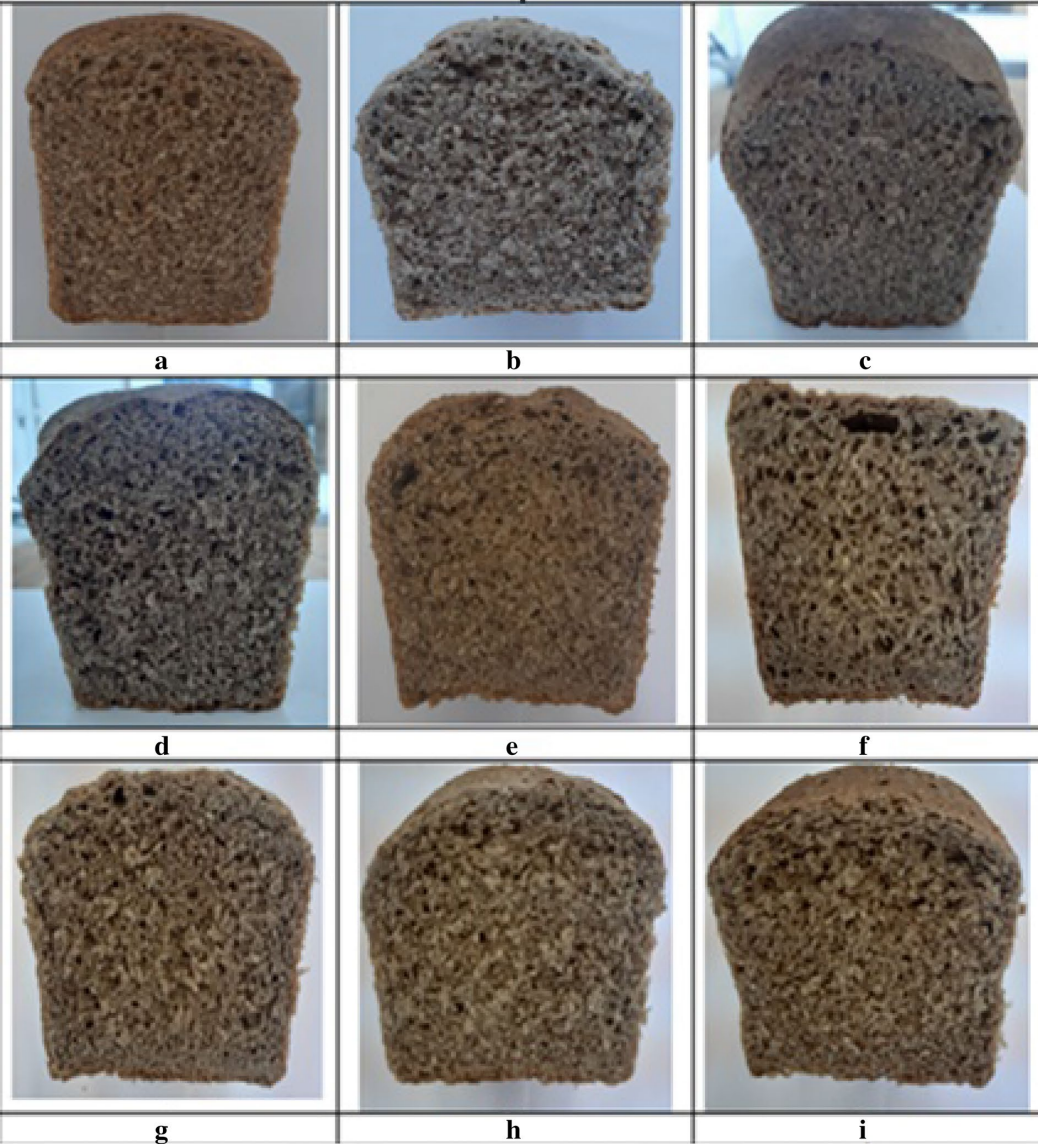
Photos of bread samples: (**a**) control sample, (**b**) sample 1, (**c**) sample 2, (**d**) sample 3, (**e**) sample 4, (**f**) sample 5, (**g**) sample 6, (**h**) sample 7, (**i**) sample 8.

**Figure 2:**
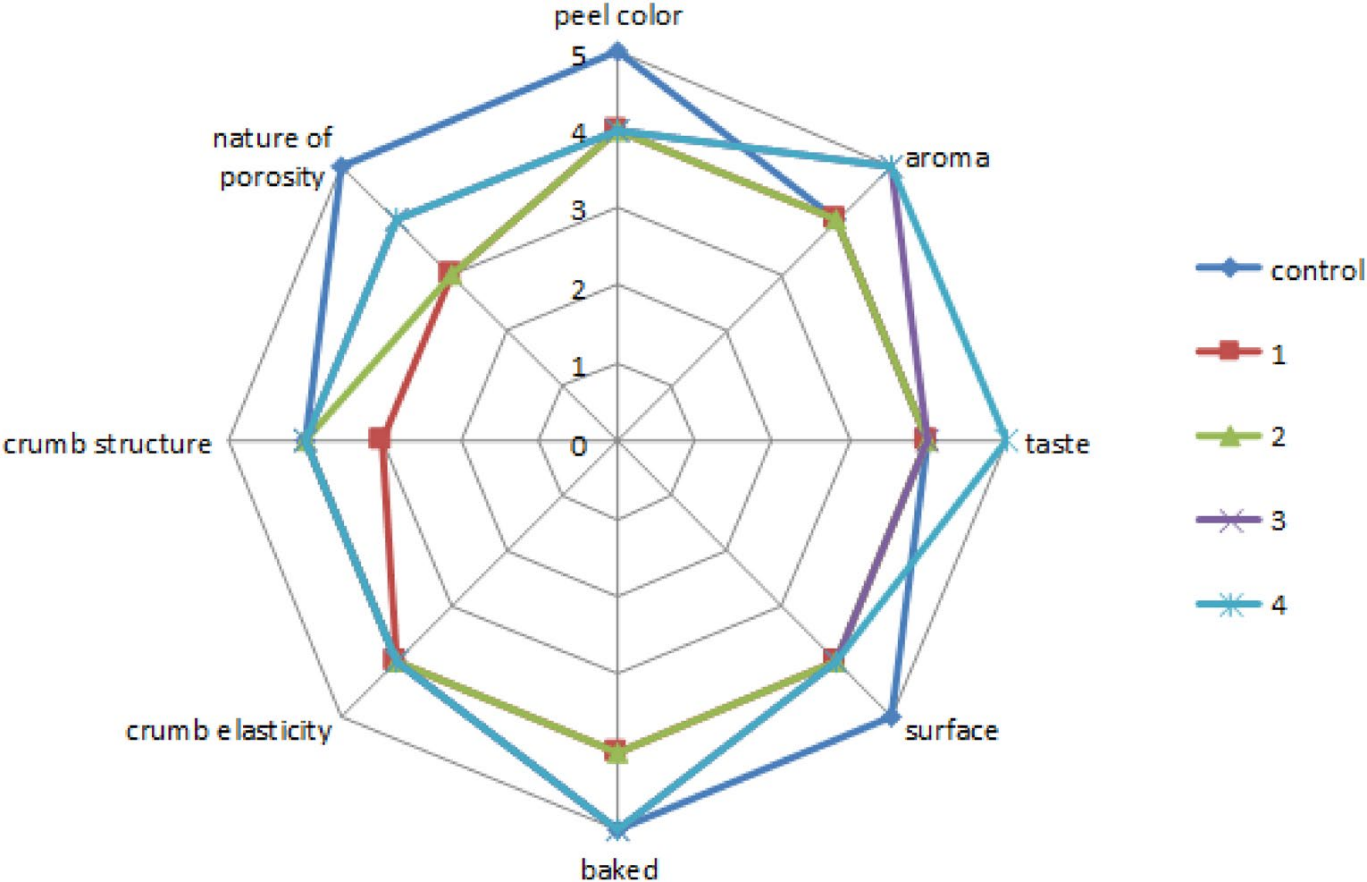
Organoleptic indicators of samples 1–4 in comparison with the control.

**Figure 3:**
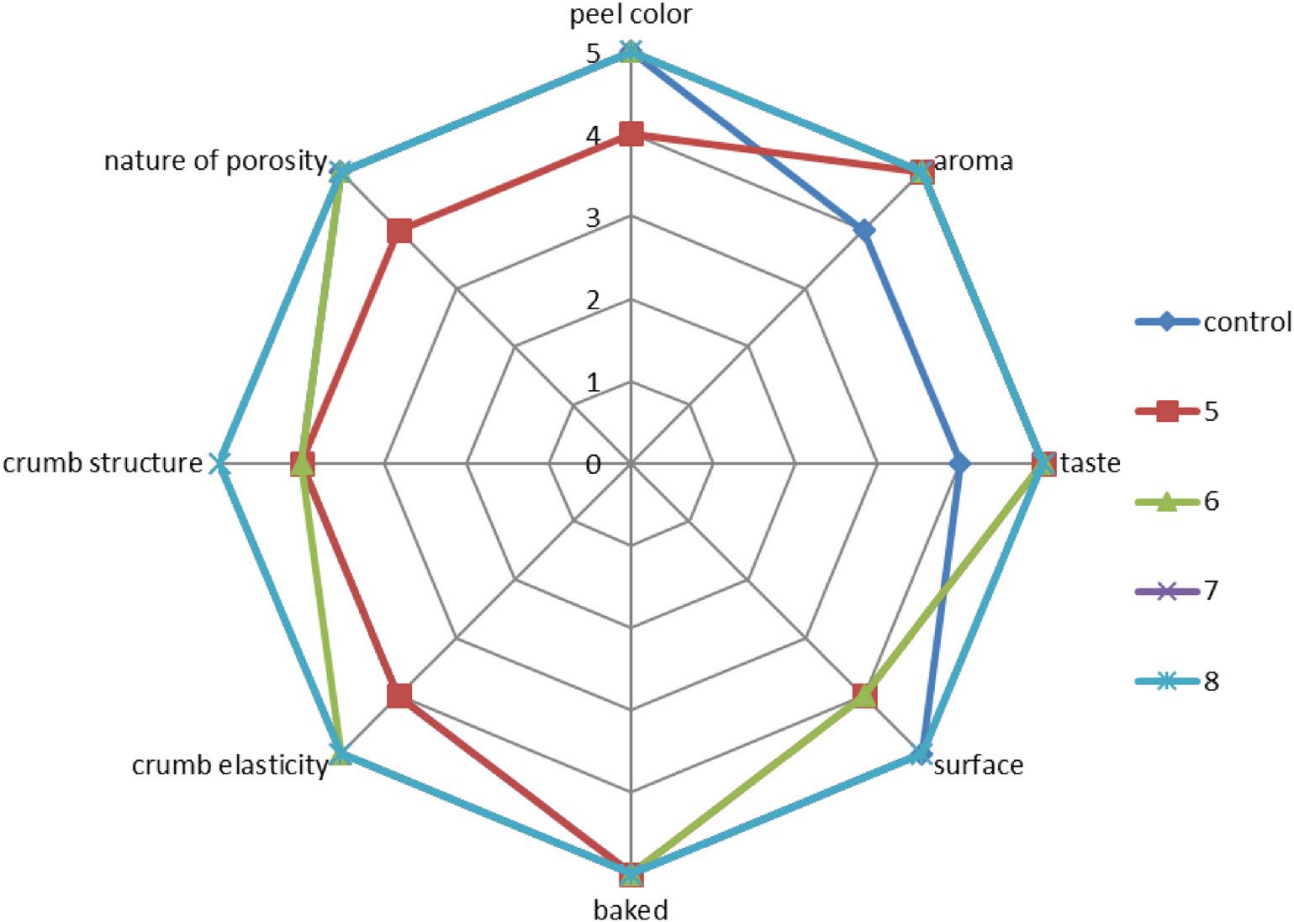
Organoleptic indicators of samples 5–8 in comparison with the control.

Figure 1 shows the types of bread products obtained, Figs. 2 and 3 show their organoleptic indicators on a five-point scale and Table 4 shows the results of these studies.

**Table 4:** Wholegrain bread made from class 3 wheat.

No.	Factors					Physical and chemical indicators of bread										
	X_0_	X_1×10_4, unit/mg		X_2_, atm	X_3_, min	y_1_, %	y_2_, %	y_3_, %	y_4_, %	y_5_, %	y_6_, %	y_7_, deg.	y_8_, %	y_9_, %	y_10_, deg.	y_11_, cm^3^
**Control sample**						3.9	0.45	37.7	2.2	3.46	51.1	5.3	65.0	46.0	4.6	231.9
1	+		25	2.0	10	4.2	0.47	34.8	2.03	3.19	46.5	4.6	59.5	44.0	4.2	209.5
2	+		5	2.0	10	4.37	0.48	33.0	1.94	3.04	50.3	4.8	60.7	42.0	4.5	183.7
3	+		25	1.0	10	3.89	0.5	37.8	2.21	3.47	47.7	5.2	62.7	43.0	4.3	218.7
4	+		5	1.0	10	4.68	0.52	34.9	2.04	3.2	49.5	3.8	58.6	41.0	4.3	225.3
5	+		25	2.0	5	4.52	0.48	33.7	2.01	3.33	53.0	5.1	62.9	45.0	4.2	216.6
6	+		5	2.0	5	4.64	1.09	30.6	1.78	2.8	46.9	4.6	63.3	43.0	4.1	205.6
7	+		25	1.0	5	4.59	0.5	35.4	2.03	3.24	52.5	5.8	57.9	44.0	4.6	227.5
8	+		5	1.0	5	4.49	0.52	32.0	1.87	2.94	54.5	5.5	59.1	42.0	4.4	229.6

y_1_—protein, y_2_—fats, y_3_—carbohydrates, y_4_—ash content, y_5_—fibre, y_6_—dough moisture, y_7_—dough acidity, y_8_—porosity, y_9_—bread moisture, y_10_—bread acidity, degree, y_11_—bread volume.

Organoleptic indicators were examined on a five-point scale. The assessment was carried out by independent experts aged 18 to 60 years. The average values of the obtained sensory data are shown in Figs. 2 and 3.

From the data obtained from photographs and organoleptic assessment (Figs. 1, 2, 3), the following conclusions can be drawn: in comparison with the control sample, samples 6–8 differ favourably in almost all indicators. Samples 1–5 are less attractive compared to the control sample in terms of colour, crust surface, crumb, etc. and numbers 6–8 are even more attractive than the control. All investigated indicators were rated on a five-point scale, while the control sample was rated at four points for many indicators. The taste, aroma, baking, crumb and pore structure of the bread products were better than those of the control sample. Table 4 shows the physicochemical and rheological indicators of the finished bread.

From Table 4, it can be concluded that, in many respects, breads made with ion-ozone cavitation treatment have more attractive properties in terms of protein content, fat and bread volume. The most attractive were numbers 3, 5, 6, 7 and 8. Numbers 4, 6 and 7 have the best protein content, number 6 the best fat content, and numbers 3 and 7 the best carbohydrate content. In terms of ash and fiber content, samples of bread products numbered 3, 4, 5, 7 showed high results. Porosity was best in numbers 5 and 6, and the best bread volume was found in numbers 3, 7 and 8. Sample 7 was found to be the most optimal treatment: it is better than many samples in terms of protein, carbohydrates, fibre, bread volume, etc.

Further, the microbiological indicators of finished bakery products were investigated; the results are shown in Table 5.

**Table 5:** Microbiological indicators of finished bakery products.

No.	Number of mesophilic aerobic and facultative anaerobic microorganisms, CFU/g, no more than					
	After 12 h	After 24 h	After 48 h	After 96 h	After 168 h	After 192 h
**Control sample**	3 × 10^2^	4 × 10^2^	7.5 × 10^2^	13 × 10^2^	Solid growth	
1	3.5 × 10^2^	4.9 × 10^2^	8.0 × 10^2^	Solid growth		
2	3 × 10^2^	4 × 10^2^	7.5 × 10^2^	Solid growth		
3	2.5 × 10^2^	3.7 × 10^2^	7 × 10^2^	Solid growth		
4	2.3 × 10^2^	3.5 × 10^2^	6.7 × 10^2^	10 × 10^2^	Solid growth	
5	2 × 10^2^	3 × 10^2^	6.5 × 10^2^	9.3 × 10^2^	Solid growth	
6	1.5 × 10^2^	2.5 × 10^2^	6 × 10^2^	9 × 10^2^	Solid growth	
7	1.5 × 10^2^	2.5 × 10^2^	6 × 10^2^	8.5 × 10^2^	Solid growth	
8	1.8 × 10^2^	2.7 × 10^2^	7 × 10^2^	9 × 10^2^	Solid growth	

From Table 5, it can be seen that the ion-ozone cavitation treatment of samples 4–8 made it possible to obtain more and more attractive bread samples from the point of view of microbiology. Number 8 represents the degradation assessment, because the growth of microorganisms began to increase. Thus, the most attractive in terms of microbiological indicators are numbers 6 and 7, as they have the lowest indicators. The number of mesophilic aerobic and facultative anaerobic microorganisms in each bread sample was observed up to 192 h. The best option is bread sample 7: after 48 h, there were 8.5 × 10^2^ mesophilic aerobic and facultative anaerobic microorganisms on it; its shelf life is up to 4 days.

## Discussion

In fact, good progress has been made in the production of bread products over the past 10 years. Most of them are dedicated to improving quality, chemical and biochemical composition^43–45^. Also known are various methods of mechanical loosening of the dough by churning a part of it, which consists in the following: part of the dough (in a relatively liquid and cold state) is knocked down within 5 min in a special beater of strong and heavy construction. After a break, the knocked down mass is fed into an ordinary kneading machine, in which the dough is kneaded, which then goes to cutting and baking^46,47^.

However, these methods were used only for long-term dough preparation and did not find application in industry.

An analogue of the technique and technology of bakery products with a reduced production cycle is the development of a functional whipped bread technology from wheat flour by scientists of the Voronezh State Technological University of Engineering Technologies. The essence of the developed technology is as follows: at the first stage, the preparation of raw materials for production and dosing are carried out, at the second stage, the components are mixed for 9–10 min at a kneading body rotation frequency of 5 s^−1^ in an XBA mixing machine, at the third stage, the dough is churned under a pressure of 0.40 MPa for 10 min at a kneading body rotation frequency of 10 s^−1^ in a hermetically sealed 2-chamber churning unit made of first grade wheat flour and additional raw materials according to the recipe, at the fourth stage, the kneaded dough is divided and sent for baking, at the fifth stage, packaging and storage are carried out^48, 49^.

After conducting numerous scientific experiments, which included assessing the quality of grain, flour, dough and finished products by organoleptic, physico-mechanical, biochemical, rheological and microbiological indicators, we came to the following conclusions:

- the use of the ion-ozone cavitation method of treatment has been proven to lead to an improvement in performance, but there is a nuance: it depends on the treatment mode and ion-ozone concentration. Thus, the most attractive in all respects are samples 6 and 7. They are attractive in terms of microbiological, physicochemical and rheological indicators. They have good elasticity, crumb and surface structure, and bread volume;
- the use of ion-ozone cavitation treatment allows the dough preparation time to be reduced, because all bread samples were prepared according to the same technology, but in comparison with the control sample, as well as numbers 1–3, the rest of the samples, especially 6–8, turned out to have good rheological properties when baked. Thus, we can draw conclusions about the prospects of this method of dough preparation;
- the ion-ozone cavitation method is capable, depending on the mode of processing and concentration, of having a positive effect on the quality of bread without losing consumer interest. This finding opens up a whole network of opportunities for researchers as well as manufacturers.

The results of the study showed that processing option 7 (ion-ozone treatment at a concentration of 0.0025 units/mg and pressure of 1.0 atm, for 1 min) is optimal and so is recommended for processing flour and obtaining highly effective bread from it.

As a result, it was found that when treated with 0.0025 units/mg ion-ozone, at a pressure of 1.0 atm for 1 min, the dough and the bread obtained from it are of high quality in comparison with the samples obtained by the control method and those obtained by other modes.

Thus, the duration of the technological process of bread production is reduced by two to three times, the number of pieces of equipment is reduced due to the elimination of fermentation and proofing processes (bowls, fermentation tanks, proofing cabinet), the bread yield is increased by 14–18% and labour productivity is increased by more than two to three times. In this regard, the research results are extremely relevant for the whole world; they will make a huge contribution to the development of science and technology in the bakery industry, which will have a direct impact on the national security of the country.

Thus, we consider the obtained results to be very valuable and require attention and refinement. We plan to conduct more extensive studies on the processes, economic value and mathematical analysis of the results obtained.

## Materials and methods

### Raw materials and supplies

The following raw materials were used for the manufacture of the test samples: class 3 soft wheat flour (1 sample), acquired from LLP “Mibeko” (Kostanai, Kazakhstan), edible salt (GOST R 51574-2000)^50^ and drinking water (SanPiN 2.1.4.1074-01)^51^.

The main stage in preparing dough by a mechanical method is knocking down the semi-finished product using cavitation (excess pressure). The aim of the research was to investigate the possibility of obtaining whipped bread from low-quality class 3 flour by mechanical loosening under pressure in an ion-ozone cavitation unit.

### Methods of determination

They were studied physical and biochemical properties, amino acid content and grain protein. Also defined the organoleptic, physico-chemical and microbiological properties of the indicators of bread.

Studies to determine the physico-biochemical properties of grain, amino acids and proteins were carried out at the basis of the Almaty Technological University, and the production of finely ground and whole flour from different classes of soft wheat and bread baking using the accelerated test method was done at the Voronezh State University of Engineering Technologies.

Physico-biochemical and biochemical properties were determined in wheat grain. The moisture content of the flour was determined by the accelerated method, according to GOST 9404-88^52^. The content of raw gluten was controlled according to GOST 27839-88^53^. The quality of raw gluten was determined by measuring its elastic properties, according to GOST 27839-88^53^. The mass fraction of protein was determined according to GOST 10846-91^54^, the fat content was determined according to GOST 29033-91^55^ and the mass fraction of fibre was determined according to the Wend method. The vitreous nature of wheat grains was determined on a diaphragmoscope according to GOST 10987-76^56^.

The experiment was carried out in threefold repetition. Taking into account the presence in many equations of significant coefficients of pair interactions (i.e., non-linearity of the objective function and quality assessment criteria), the search for optimal processing modes was carried out using non-linear programming methods—Newton’s method included in the ‘Search for a solution’ procedure in the MS Office Excel package.

### Devices and installations

**Figure 4:**
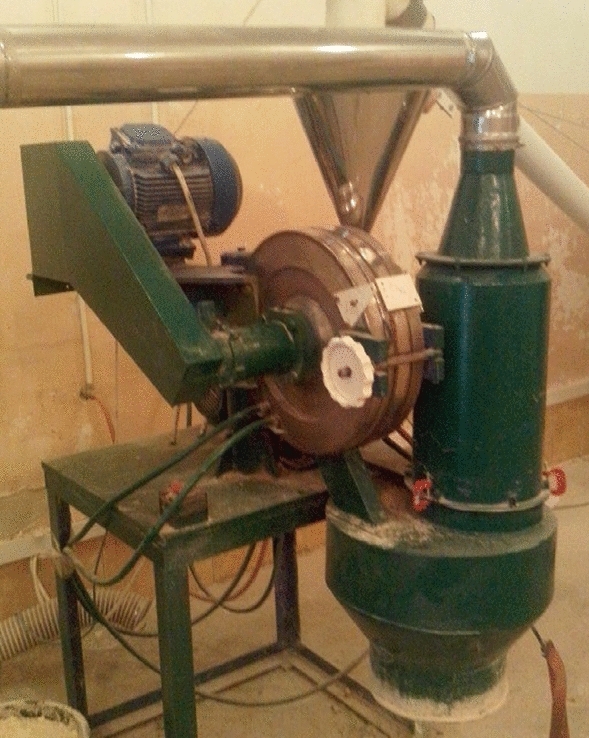
Appearance of the disintegrator: 1—loading funnel, 2—working chamber with grinding disks, 3—unloading hole, 4—filter, 5—electric motor.

Flour from whole-ground wheat grain was obtained by disintegration-wave grinding on a disintegrator (Voronezh State University of Engineering Technologies, Russia) (Fig. 4). Wheat grains are fed into the working chamber 2 through the feed funnel 1, which is equipped with a grate for additional removal of weed particles exceeding the size of the grains.

The electric motors 6 drive the grinding disks 3 and stand in such a way that the movement of the magnetic disks occurs to meet each other. Due to this design feature, a high number of revolutions (18,000–25,000 rpm), and a small gap between the pins of the grinding disks, the grains are crushed with a higher degree of dispersion than other types of mills, which allows obtaining a high quality product.

In addition, in a very short period of time, synchronized conditions for the interaction of the field and matter at the atomic-molecular level arise in the chamber. This causes positive changes in the physicochemical state of the surface structure, which is the mechanical activation of the feedstock.

Through the inlet, the gap of which is regulated depending on the selected raw material in accordance with its size, the mass enters the working chamber, where it is ground. Ready flour through the discharge opening 4 is fed into the bag.

Scientists at the Almaty Technological University have developed ion-ozone cavitation units. Crops were processed and their physicochemical, microbiological, biochemical and other qualitative and quantitative indicators were examined before and after processing. The results of research and materials on ion-ozone and cavitational treatment have been presented in previous articles^57,58^.

**Figure 5:**
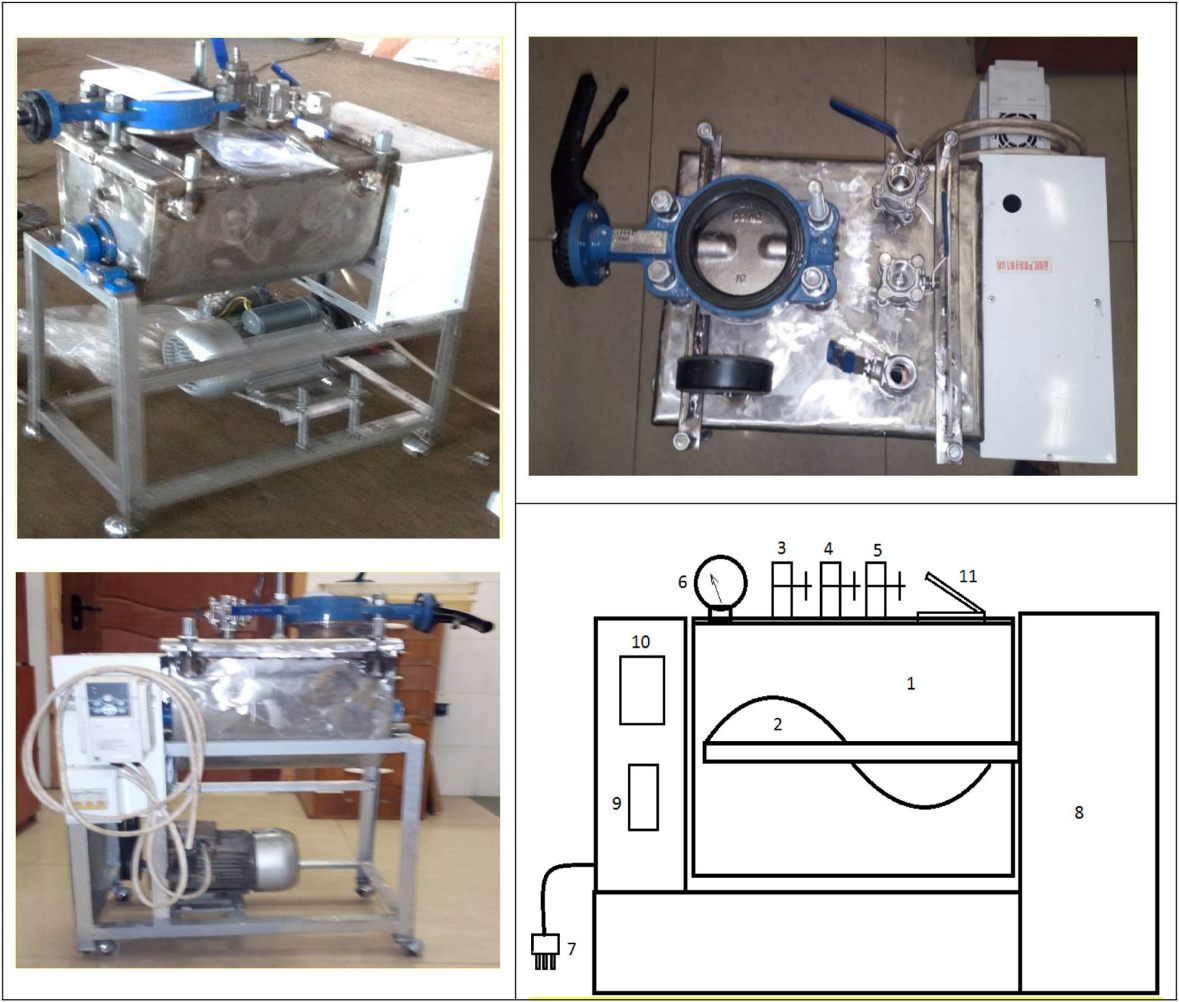
Ion-ozone cavitation unit for dough preparation with an accelerated cycle: 1—container for dough preparation; 2—bubble shaft; 3—flour dispenser; 4—dispenser for water and other components; 5—branch pipe for supplying ozone, or molecular or atomic ions of oxygen in the air; 6—pressure detection sensor; 7—electric wire; 8—electric drive; 9—fuses; 10—electrical converter; 11—cavitator lever.

The dough was made in a laboratory ion-ozone cavitation unit, which is shown in Fig. 5.

The dough was obtained by mechanical loosening under pressure in an ion-ozone cavitator installation for preparing dough with an accelerated cycle, developed by scientists at the Almaty Technological University (Mayemerov M.M. and Iztaev A.I.).

The ion-ozone cavitation unit for preparing dough with an accelerated cycle is intended for use in bakery and grain-receiving plants.

### Recipe and dough preparation modes

The main advantages of this installation are the range of churning parameters (rotational speed of the kneading body, the pressure supplied), the smoothness of their regulation, the possibility of obtaining churned masses of various compositions and air, and ease of installation, design and maintenance.

Whipped yeast dough was prepared for a laboratory ion-ozone cavitation unit, at 55–56% humidity according to the recipes and modes shown in Table 6.

**Table 6:** Recipe and dough cooking modes for whipped yeast bread made from grade 3 flour.

No.	Raw materials and process indicators	Raw material consumption and process parameters
1	Wheat flour, class 3, kg	10.0
2	Yeast for dough, kg	0.25
3	Table salt, kg	0.15
4	Vegetable oil, kg	0.02
5	Water, l	By calculation
6	Dough moisture, %	50–55
7	Mixing duration, min	5–7
8	Churning duration, min	2–3
9	Cavitation (overpressure), atm	1–2
10	Kneading body rotation frequency, Hz	15–30

In order to reduce the time and remove some stages of the technological process of preparing dough when baking bread and bakery products, as well as to increase the biological value of the processed products, they were produced in an ion-ozone cavitation unit using ion-ozonated structured water charged with ozone, atomic or molecular ions and their mixtures, and in accordance with the technological process with cavitation. This process helps to reduce production costs, ensure high processing speed and guaranteed product quality, ecological and biological purity, minimum energy consumption, reliability and ease of use. When improving the process of baking bread and bakery and other products, the most important thing is to obtain an environmentally friendly food product of improved quality by a simpler and shorter technological process.

The ion-ozone cavitation unit for dough preparation corresponds to the apparatus used in production. All units and parts are manufactured in accordance with the requirements of the technical assignment and other regulatory documents.

The ion-ozone cavitation unit for dough preparation is an installation consisting of units and mechanisms of control, regulation and protection, combined into one whole by a common ion-ozone cavitator, which ensures the safety of the service personnel.

All units and parts of the working bodies are made of food-grade stainless steel. The electric motor has a frequency regulator of shaft rotation speed. The laboratory ion-ozone cavitation unit for dough preparation has productivity of 50 kg/h, dimensions of 1000 × 800 × 800 mm and an installation weight of 50 kg.

In the design of the laboratory ion-ozone cavitation unit, standard materials, profiles and electrical devices were used to prepare the dough. The controls of the laboratory ion-ozone cavitation unit for dough preparation and its technological organs fulfil ergonomic and aesthetic requirements. All electrical wiring is made in accordance with the requirements of GOST.

The kneading machine works as follows: the recipe components of the dough are fed through the inlet into the kneading body of the batch kneader, in which the kneading body is installed, driven by an electric motor by means of a speed variator. At the end of loading, the kneading body of the kneading machine is hermetically closed with a lid and the dough is kneaded for 3–5 min at a kneading body rotation frequency of 5 s^−1^. Then, ion-ozonized cavitation air is fed into the kneading chamber under a pressure of 0.20, 0.40 or 0.60 MPa and the dough is beaten for 3–10 min at a kneading body rotation frequency of 2–3, 4–5 or 7–8 s^−1^. When the recipe components are churned, the dough mass is saturated with air. The dough prepared in this way is a foamy mass with stable physicochemical characteristics. The process of churning dough from grade 1 and 2 wheat flour was investigated at 0.20, 0.40 and 0.60 MPa and a kneading body rotation frequency of 2–3, 4–5 and 7–8 s^−1^ for 2–3, 4–5 and 7–10 min and without compressed air supply.

Baking of bread products was carried out in baking ovens with parameters that provide optimal technological conditions and baking mode. The duration of baking at a baking chamber temperature of 220 °C was 25–30 min, depending on the weight of the product.
